# Designing Tunable Paper-Based Colorimetric Sensor for Precise Detection of Hydrogen Peroxide Vapor

**DOI:** 10.1021/acsomega.5c01380

**Published:** 2025-08-01

**Authors:** Rayhan Hossain, Allen Apblett, Nicholas F. Materer

**Affiliations:** † Department of Natural Sciences, 107 Science Faculty Center, University of Michigan, Dearborn, Michigan 48128, United States; ‡ Department of Chemistry, 316 Physical Science, 7618 Oklahoma State University, Stillwater, Oklahoma 74078, United States

## Abstract

Detection of hydrogen peroxide (H_2_O_2_) vapor remains a significant challenge for conventional sensing technologies, despite its significance in applications such as the detection of improvised explosive devices (IEDs). Herein, we report a novel, highly sensitive colorimetric sensor system capable of detecting H_2_O_2_ vapor at concentrations as low as parts-per-billion (ppb). The sensor is based on a cellulose microfibril network, derived from paper towels, which provides a versatile and tunable substrate for the incorporation of Ti­(IV) oxo complexes. These complexes selectively bind to H_2_O_2_, forming a Ti­(IV)-peroxide coordination complex that induces a prominent chromatic shift from colorless to bright yellow, with an absorption maximum at approximately 400 nm. This complexation-driven color transition exhibits exceptional selectivity for H_2_O_2_, with no detectable color change in the presence of water, oxygen, common organic solvents, or other chelating agents. The sensor is designed for single-use and is inherently low-cost, providing a simple yet effective approach for H_2_O_2_ vapor detection. Additionally, the system highlights the potential of cellulose-based nanofibril materials in advancing colorimetric sensing platforms. By reducing the fiber dimensions, the available surface area for interaction with gaseous analytes is significantly enhanced, thus improving the sensitivity and overall performance of the sensor. This work not only demonstrates the feasibility of an efficient paper-based sensor for H_2_O_2_ vapor detection but also opens avenues for further exploration into nanostructured materials for the development of next-generation sensing technologies.

## Graphical abstract

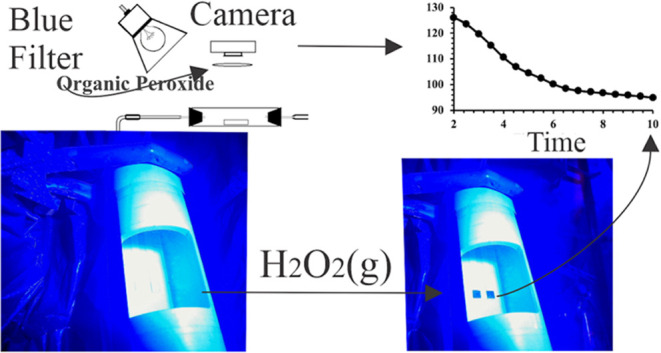

## 1 Introduction

The detection of hydrogen peroxide (H_2_O_2_) vapor is of considerable importance across a broad range of applications, from environmental monitoring and industrial safety to the detection of chemical agents, such as those employed in improvised explosive devices (IEDs). Despite its significance, the real-time, sensitive detection of H_2_O_2_ vapor remains a formidable challenge, primarily due to the low vapor pressure and low concentration of H_2_O_2_ in ambient environments, which necessitates highly sensitive and selective sensors. Conventional detection techniques, such as chemiluminescence, electrochemical sensors, and spectroscopic methods, often suffer from limitations in terms of sensitivity, specificity, and cost-effectiveness, especially in low-concentration environments. These shortcomings highlight the pressing need for novel sensing platforms that combine high sensitivity, selectivity, and ease of fabrication, while also being scalable and inexpensive.^1–3^

In recent years, significant progress has been made in developing colorimetric sensors, which offer a user-friendly and cost-effective means of detecting analytes through visual changes in color. Colorimetric sensing has garnered substantial attention due to its simplicity and the ability to provide immediate, intuitive readouts without the need for specialized instrumentation. Among the various materials explored for colorimetric sensing, cellulose-based substrates have emerged as promising candidates due to their abundance, biodegradability, and inherent tunability. The highly porous nature of cellulose microfibrils provides a large surface area that can be readily functionalized with active chemical sites, making them suitable for a range of sensing applications, including gas detection.^4–6^

Cellulose-based materials have shown particular promise for environmental monitoring, where low-cost and high-performance sensors are required to detect a wide array of gaseous analytes.^7–10^ The incorporation of metal ions or metal oxide complexes with cellulose matrices has been explored as an effective strategy for enhancing sensitivity and selectivity for specific gases.^11,12^ Among these, titanium-based complexes, particularly titanium­(IV) oxo species, have demonstrated excellent affinity for H_2_O_2_, enabling the development of sensors with high selectivity for hydrogen peroxide detection.^13–15^ The interaction between titanium­(IV) and H_2_O_2_ leads to the formation of a Ti­(IV)-peroxide coordination complex, which induces a color change that can be readily observed using simple optical techniques (see Figure 1). This phenomenon has been leveraged in a variety of sensing platforms, including paper-based sensors, for the detection of trace amounts of H_2_O_2_.^16–18^

**Figure 1:**
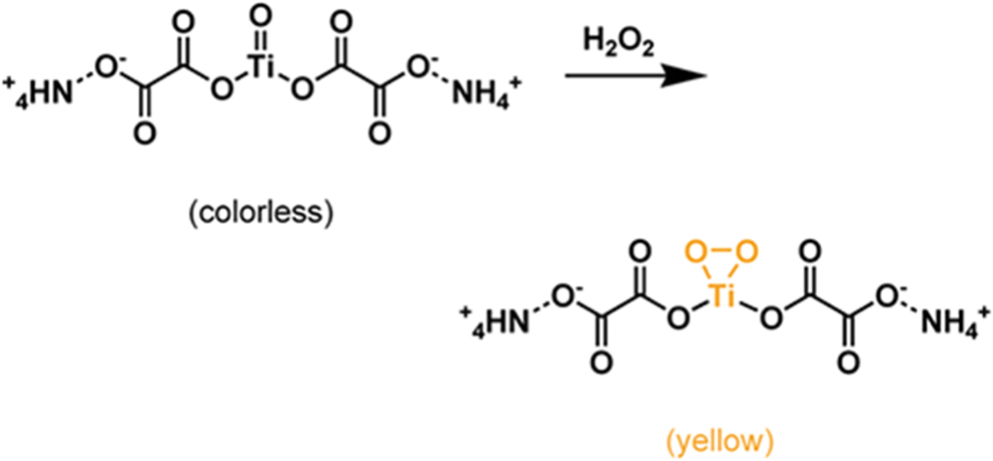
Suggested colorimetric detection of H_2_O_2_ based on complexation with a Ti­(IV)-oxo moiety. Upon exposure to H_2_O_2_, the initially colorless ammonium oxo-titanium complex forms a yellow-colored peroxo–Ti­(IV) species through coordination of a bidentate peroxide ligand to the titanium center. The color change serves as a visual indication of H_2_O_2_ presence.

In this work, we introduce a novel, highly sensitive colorimetric sensor system designed for the detection of H_2_O_2_ vapor at concentrations as low as parts-per-billion (ppb). The sensor is based on a cellulose microfibril network, derived from paper towels, which serves as a versatile substrate for the incorporation of titanium­(IV) oxo complexes. These titanium-based complexes exhibit a strong and selective affinity for hydrogen peroxide, forming a Ti­(IV)-peroxide coordination complex upon exposure to H_2_O_2_. This coordination leads to a pronounced colorimetric change from colorless to bright yellow, with an absorption peak at approximately 400 nm. The sensor’s performance is characterized by its exceptional selectivity for hydrogen peroxide over other potential interfering substances, including water, oxygen, common organic solvents, and other chelating agents. This high selectivity is a critical attribute for the development of a practical and reliable sensor for H_2_O_2_ vapor, particularly in complex and dynamic environments.^19–21^

The incorporation of cellulose microfibrils, derived from inexpensive and widely available paper towels, provides a low-cost and scalable platform for the development of these sensors. Furthermore, by reducing the fiber dimensions of the cellulose network, the available surface area for interaction with gaseous analytes is significantly enhanced, resulting in improved sensitivity and faster response times.^22–24^ This work not only demonstrates the feasibility of an efficient paper-based sensor for H_2_O_2_ vapor detection but also showcases the potential of nanostructured cellulose materials in advancing the field of colorimetric sensing technologies. By leveraging the unique properties of cellulose and titanium oxo complexes, we present a promising new direction for the development of next-generation, low-cost, high-performance sensors.^25–28^

To optimize the Ti­(IV)-peroxide coordination complex, various factors were systematically studied, including the concentration of titanium precursor, the peroxide-to-metal ratio, and the reaction time under different temperature conditions. By adjusting these variables, we were able to fine-tune the formation of the Ti­(IV)-peroxide complex, ensuring its stability and enhancing its reactivity toward H_2_O_2_ vapor. Additionally, the interaction between the complex and the cellulose matrix was optimized by altering the cellulose microfibril dimensions, which provided a greater surface area for the interaction with H_2_O_2_ and improved the sensor’s sensitivity and response time.

Recent advances in vapor-phase hydrogen peroxide detection have primarily relied on rigid sensor platforms or complex nanomaterial-based systems, which often require specialized fabrication processes and instrumentation for signal readout. In contrast, this work introduces a simple yet highly sensitive paper-based colorimetric sensor that is flexible, disposable, and cost-effective. By carefully tuning reagent composition and optimizing the interaction between the sensing matrix and peroxide vapor, our design achieves a remarkably low detection limit of 0.04 ppb. The clear visual color change enables both qualitative and quantitative analysis using accessible tools. These features make the sensor particularly attractive for real-time, on-site forensic applications, including the detection of trace peroxide-based explosives or chemical residues in crime scene investigations.

In recent years, various materials have been explored for colorimetric sensing, each with distinct advantages and limitations. Traditional cellulose-based sensors offer biodegradability, cost-effectiveness, and ease of functionalization, making them attractive for environmental and healthcare applications. However, these sensors often suffer from limited detection ranges and environmental sensitivity, which restricts their performance in certain sensing environments. In contrast, other materials such as polymer-based membranes, metal–organic frameworks (MOFs), and nanocomposites have been investigated to address these shortcomings. Polymer membranes, for instance, provide improved stability but often lack the sensitivity and selectivity required for accurate detection.^29^ MOFs and nanocomposites offer higher sensitivity and tunability, yet challenges remain in terms of scalability, cost, and integration into practical devices.^30^ Recent advances in cellulose-based sensors have sought to mitigate these limitations, with improvements in detection limits and stability achieved through the incorporation of functional groups or hybrid materials.^31^ In this work, we have developed a cellulose-based sensor that significantly enhances detection limits and extends the sensing range compared to previous designs. Our sensor demonstrates improved sensitivity and environmental stability, offering a more reliable and versatile platform for colorimetric detection in diverse applications.

In the following sections, we will describe the synthesis of the sensor materials, the underlying sensing mechanism, and the sensor’s performance characteristics. We will also discuss the potential applications of this sensor in a range of critical fields, from environmental monitoring to defense and security, and highlight opportunities for future research and development aimed at enhancing the capabilities of cellulose-based colorimetric sensors.^32–34^

## 2 Experimental Methods

### 2.1 Materials

Ammonium titanyl oxalate monohydrate and all other chemicals were obtained from Fisher Scientific and used without further purification. The paper substrates used were SAFECHEM Tork Advanced Perforated Towels (white, HB9201). Deionized (DI) water was used in all aqueous preparations. All chemicals were of analytical grade.

### 2.2 Instrumentation and Characterization

Ultraviolet–visible (UV–vis) absorption spectra were measured using a PerkinElmer Lambda 1050 spectrophotometer. Optical microscopy images were captured with a Leica DMI4000B inverted microscope equipped with a high-resolution CCD camera. A CR-10 color reader, obtained from Konica Minolta Sensing Americas, Inc., was employed for color analysis (accuracy: ±0.1). Vapor exposure experiments utilized a mini fan (40 mm, 12 V DC, 6500 rpm) sourced from Radio Shack. FTIR spectra were collected using a Thermo Scientific Nicolet iS50 FTIR spectrometer in attenuated total reflectance (ATR) mode, with a resolution of 4 cm^–1^ and 32 scans per spectrum in the range of 4000–500 cm^–1^. Surface chemical composition and oxidation states were analyzed using a Kratos AXIS Ultra DLD XPS system with a monochromatic Al Kα X-ray source (1486.6 eV). Survey and high-resolution spectra were collected under ultrahigh vacuum (UHV) conditions, and binding energies were referenced to the C 1s peak at 285 eV.

### 2.3 Paper Sample Preparation

Paper samples were prepared by drop-casting 100 μL of an aqueous solution of ammonium titanyl oxalate onto a 2.5 × 2.5 cm^2^ piece of paper towel, followed by vacuum drying at room temperature for 1 h. To achieve varying molar loadings of titanyl salt, stock solutions of ammonium titanyl oxalate were prepared at different concentrations: 0.001, 0.005, 0.01, 0.02, 0.05, 0.1, 0.2, 0.4, 0.8, and 1.0 mol/L.

The inherent homogeneity of the fibrillar structure of the paper towel enabled rapid absorption and uniform distribution of the solution across its matrix. For precise and consistent sample preparation, 100 μL of the solution was evenly drop-cast at nine points (3 × 3 grid) on the paper surface, ensuring uniformity in titanyl salt distribution across the entire area. This homogeneity was confirmed by the consistent color density observed upon exposure to hydrogen peroxide vapor.

### 2.4 Vapor Sensing Test

For vapor sensing measurements at a fixed hydrogen peroxide vapor pressure, the test was conducted by suspending the loaded paper towel in a saturated vapor environment (230.4 ppm). This vapor was generated above 10 mL of a 30 wt % H_2_O_2_ solution contained in a sealed 50 mL vial. The resulting yellow coloration, developed over specific time intervals, was quantified using a CR-10 color reader.

For measurements at a fixed titanyl salt loading, approximately 1 L of H_2_O_2_ solution, diluted to various concentrations, was placed in a 10 L sealed container and allowed to equilibrate for 12 h to achieve steady-state vapor pressure. The equilibrium vapor pressures corresponding to specific H_2_O_2_ solution concentrations were derived from literature data.

During the sensing tests, the prepared paper towel samples were positioned approximately 0.5 cm from the center of a fan (12 V, 6500 rpm), which was suspended within the sealed container approximately 20 cm above the solution surface. The fan directed vapor toward each sample for varying exposure times, as detailed in Figure 3. After exposure, samples were removed for color measurements.

To achieve varying low concentrations of H_2_O_2_ vapor, the commercial 30 wt % solution was diluted with deionized water at ratios of 1:1000, 1:500, 1:300, 1:200, 1:100, 1:75, 1:50, 1:25, and 1:10. These dilutions generated saturated equilibrium vapor pressures of 0.1, 0.2, 0.3, 0.5, 1.0, 1.3, 1.9, 4.0, and 10.5 ppm, respectively.

## 3 Results and Discussion

The visualization of the microstructural interaction between ammonium titanyl oxalate monohydrate (ATO) and hydrogen peroxide (H_2_O_2_) is a sophisticated representation of the molecular dynamics involved in this interaction, based on the principles of computational chemistry and molecular modeling. This type of visualization relies on advanced simulation techniques such as density functional theory (DFT) or molecular dynamics (MD) simulations to provide a high-resolution depiction of the molecular arrangement and interaction mechanisms at the atomic scale.

In the case of ammonium titanyl oxalate monohydrate, the molecular structure typically consists of a central titanium ion (Ti^4+^) coordinated to the oxalate ligand (C_2_O_4_^2–^) and a water molecule (H_2_O) in a monohydrate form, which influences its solubility and reactivity. The interaction with hydrogen peroxide introduces additional complexities in the form of protonation and coordination effects due to the presence of the peroxide group (O_2_^2–^). This interaction is critical for understanding the potential catalytic or redox properties of ATO in reactions where H_2_O_2_ serves as an oxidizing agent.

Visualization (see Figure 2) employs standard atomic coloring conventions to facilitate the identification of specific atoms and functional groups. For instance, carbon atoms are typically depicted in gray or black, oxygen atoms in red, hydrogen atoms in white, titanium in blue, and nitrogen in purple. This color coding enhances the clarity of the structural analysis, making it easier to identify the specific sites of interaction between the ammonium titanyl oxalate monohydrate and hydrogen peroxide.

**Figure 2:**
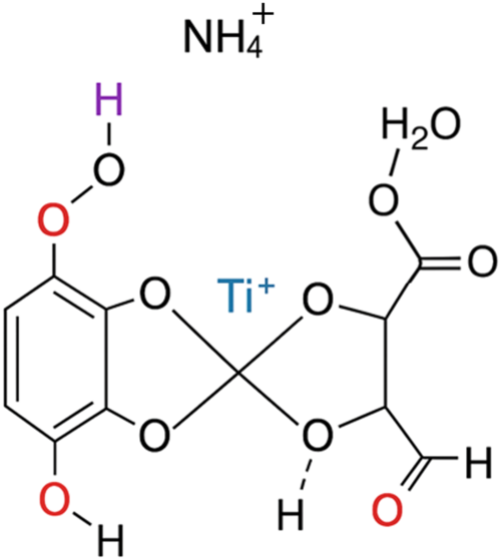
Visualization of the microstructural interaction between ammonium titanyl oxalate monohydrate and hydrogen peroxide. It accurately represents molecular interactions and uses standard atomic coloring conventions.

At the molecular level, hydrogen peroxide can coordinate to the titanium center or interact with the oxalate moiety, leading to the formation of transient species or the modulation of bond strengths, particularly in the titanium–oxygen or titanium-peroxide interactions. The visualization thus serves as a tool for studying the electron density distribution, potential reaction pathways, and the impact of these molecular interactions on the material’s reactivity, which are critical for applications in catalysis, materials science, and environmental chemistry. The computational model integrates the electrostatic forces, van der Waals interactions, and covalent bonding character, providing insights into the mechanistic steps involved in the reaction processes between ATO and H_2_O_2_.

This high-fidelity depiction of molecular interactions is crucial for designing and optimizing processes that utilize ammonium titanyl oxalate monohydrate and hydrogen peroxide, especially in fields such as heterogeneous catalysis, environmental remediation, and advanced materials development.

### 3.1 Thin Film’s Reactivity to Hydrogen Peroxide

When exposed to peroxide vapors, the films exhibit a discernible colorimetric response, consistent with prior observations from the analyzed films and test strips. The titanyl oxalate solution exhibited the characteristic yellow color shift upon exposure to H_2_O_2_ vapors.

### 3.2 Hydrogen Peroxide Reaction Kinetics with the Thin Film

There was no color change when a controlled thin film was exposed to peroxide vapors without adding any titanyl oxalate solution. Similarly, for H_2_O_2_ experiments, the image series captured under controlled conditions were analyzed using ImageJ to quantify intensity as a function of exposure duration. Utilizing ImageJ software, reflection images were extracted from the dynamic red regions of thin films, as illustrated in Figure 3. To clarify the procedure, the image series were captured under controlled conditions with consistent lighting and camera settings. Using ImageJ software, we extracted reflection images from the dynamic red regions of the thin films by applying the “Color Threshold” tool to isolate these areas. We then defined a Region of Interest (ROI) around the red regions and quantified the intensity values using the “Measure” function in ImageJ. Intensity data were collected for each exposure duration, and the values were plotted to analyze the relationship between intensity and exposure time.

Using the same techniques previously applied within the enclosed box, the experiment was conducted within a PTFE cell. The corresponding images, captured using films placed within the PTFE cell under a blue light filter to enhance intensity resolution, are presented in Figure 3. Image A represents the initial exposure to peroxide vapors, and Image B illustrates the final image postexposure.

**Figure 3:**
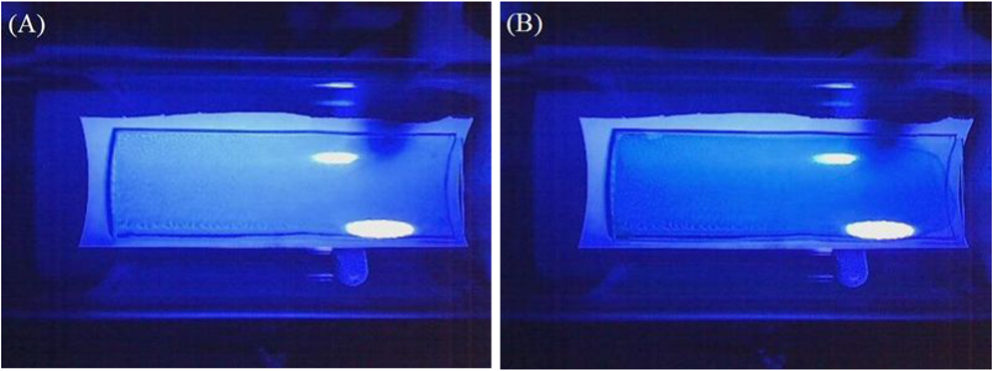
Progression of color change during exposure to H_2_O_2_ vapors.

### 3.3 The Time Progression of Color Development as Observed through UV–Vis Absorption

The data likely show a time-dependent increase in absorbance, indicative of a progressive reaction between titanyl oxalate and hydrogen peroxide (see Figure 4). The slower or faster response can provide insights into the kinetic behavior of the reaction and the mechanistic details of how H_2_O_2_ interacts with the complex. The system may eventually reach a steady state where further exposure to H_2_O_2_ does not significantly alter the absorption spectrum, implying that the reaction has either reached its limiting rate or the oxidative capacity of the H_2_O_2_ is saturated.

**Figure 4:**
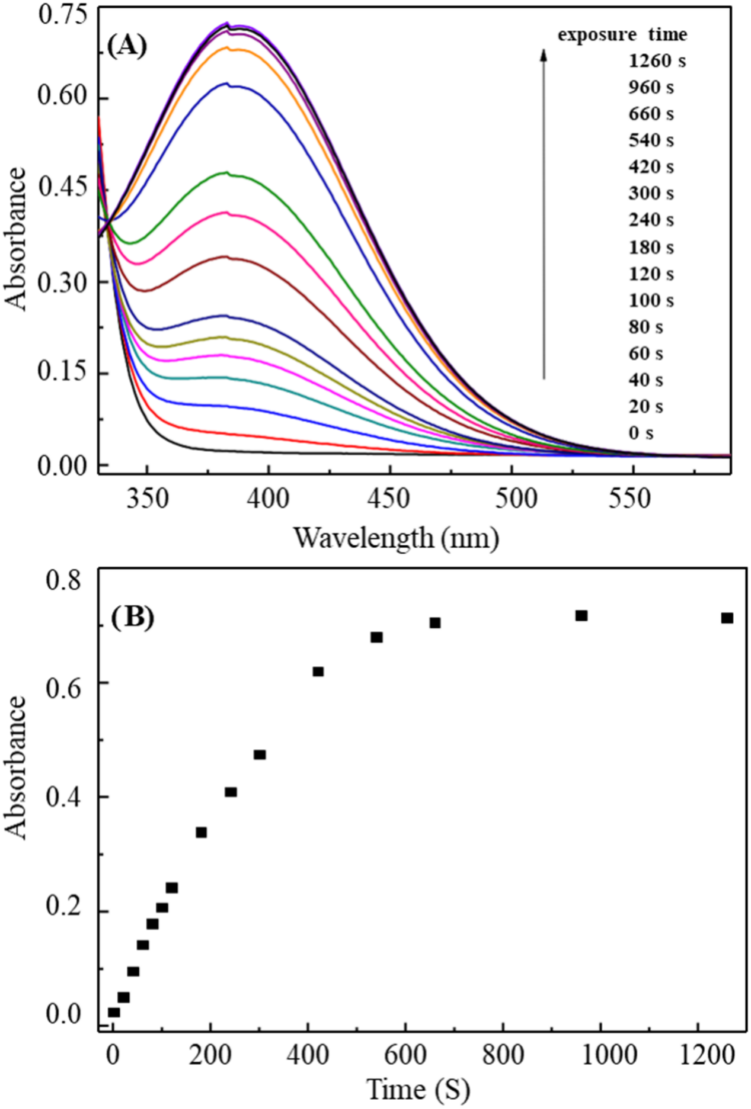
(A) UV–vis absorption spectra of the thin film of titanyl oxalate salt upon exposure to the saturated vapor of 30 wt % H_2_O_2_ solution (230.4 ppm) at various time intervals: 0, 20, 40, 60, 80, 100, 120, 180, 240, 300, 420, 540, 660, 960, 1260 s. The thin film was made by drop-casting 130 μL of 0.1 M aqueous solution of ammonium titanyl oxalate onto a quartz slide in an area of ca. 4 cm^2^. (B) Absorbance measured at the maximum wavelength (387 nm) as a function of the exposure time.

The second part of the data, absorbance at the maximum wavelength of 387 nm as a function of time, provides critical information on the kinetics of the interaction between the titanyl oxalate and hydrogen peroxide vapor. Initially, at time *t* = 0 s, the absorption at 387 nm would correspond to the absorption due to the unperturbed titanyl oxalate complex. As the exposure time increases, hydrogen peroxide begins to interact with thin film, and the absorbance increases or decreases depending on the nature of the chemical changes occurring. If the oxidation of the titanium center leads to the formation of new electronic states, a shift in the absorption spectrum (both in terms of wavelength and intensity) would be observed due to changes in the electronic structure.

The time evolution of absorbance likely follows a first-order kinetic profile or a pseudo-first-order reaction with respect to the exposure to H_2_O_2_. In this case, the absorbance would typically increase as the reaction progresses, corresponding to the formation of a new product with a different electronic configuration.

The plateauing of absorbance after a certain time (e.g., at 960 or 1260 s) suggests that the reaction has reached saturation or equilibrium. This could indicate that either the titanium center has reached its maximum oxidation state or that the concentration of hydrogen peroxide vapor has been exhausted or is no longer effective at further reacting with the thin film.

Figure 5 illustrates the reflected intensity of the entire film as a function of exposure duration. It is evident that exposure of the films to H_2_O_2_ vapors induces substantial alterations in intensity, resulting in a distinct colorimetric change. The film changes color in a manner that is similar to the first-order kinetic behavior were discussed in the article before with H_2_O_2_.

**Figure 5:**
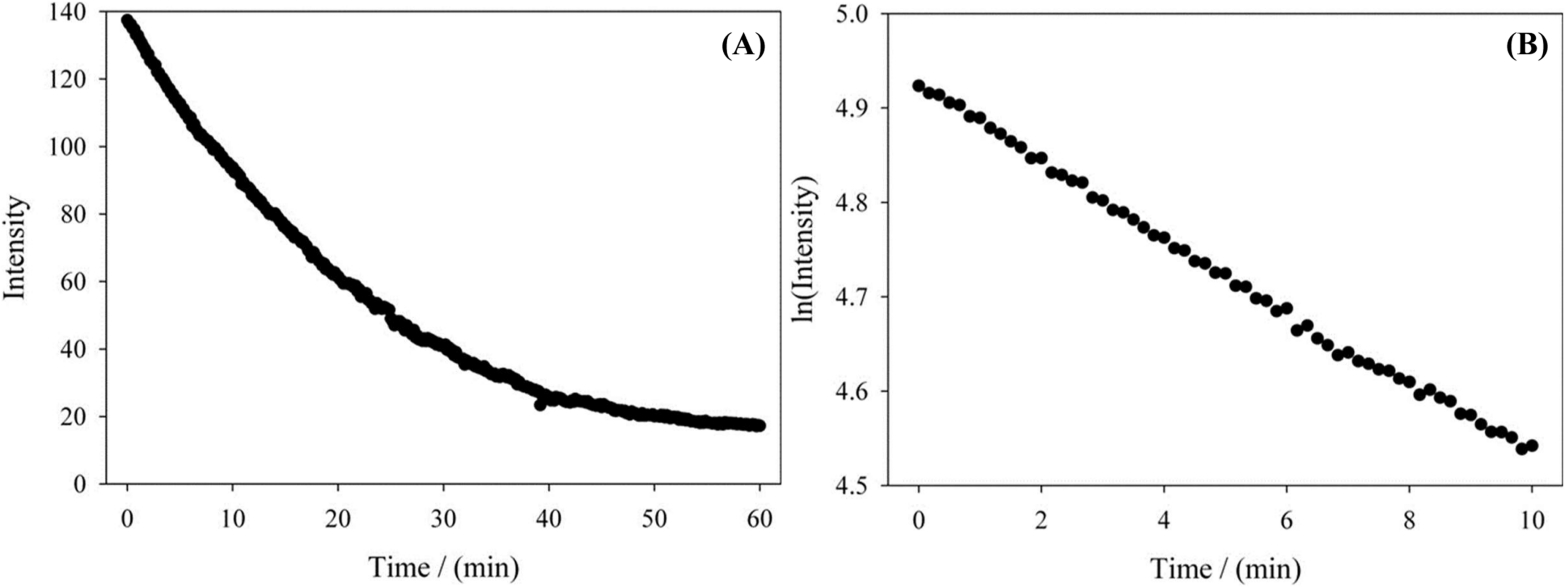
(A) Intensity versus exposure time for a paper-based substrate with H_2_O_2_ vapor exposure. (B) First-order approximation (natural log intensity vs time) for the first 10 min of exposure of hydrogen peroxide to titania coating. This approximation showed excellent linearity (*y* = −0.0398x + 4.97, *R* ^2^ = 1.00).

A reduction in reflected intensity, as depicted in Figure 5, is observed with increasing exposure time for the film. Upon exposure to peroxide vapor, the intensity exhibits an exponential decline over time. The films consistently demonstrate first-order kinetic behavior in response to H_2_O_2_ vapor diffusion, with a peroxide sensing equivalency of 0.04 ppb. Furthermore, a series of experimental runs with varying peroxide concentrations have been conducted to further validate this kinetic behavior.

The peroxide sensing equivalency value of 0.04 ppb corresponds to the sensor’s limit of detection (LOD), calculated using the standard formula: LOD = 3σ/S, where σ is the standard deviation of the baseline signal (blank) and S is the sensitivity (slope of the calibration curve). In our case, the baseline noise (σ) was determined to be 0.0025 absorbance units based on 10 replicate measurements, and the sensitivity (S) from the linear calibration plot was 0.187 absorbance units per ppb. Substituting these values yields an LOD of approximately 0.0401 ppb, which we report as 0.04 ppb.

The intensity versus exposure time plot conclusively demonstrates that the system follows first-order kinetic behavior following exposure to peroxide vapor. This was confirmed by the application of the rate constant equation, where the resulting negative slope of the plot corresponds to the characteristic exponential decay associated with first-order reactions. Linear regression analysis of the data further substantiates this observation, yielding a precise mathematical representation of the kinetic behavior.

As exposure time progresses, a saturation point is reached, at which the reflected intensity plateaus. When saturation occurs the reflected intensity also stops as there were no active titania materials remaining to interact with peroxide vapor. Once this saturation threshold is achieved, no additional chemical interaction occurs, effectively halting the intensity change.

The FTIR spectrum (see Figure 6) of ammonium titanyl oxalate monohydrate interacting with hydrogen peroxide offers comprehensive insights into its molecular structure and bonding dynamics. The peaks observed in the 450–800 cm^–1^ region are primarily attributed to Ti–O stretching vibrations within the titanyl (TiO) framework. These vibrations are characteristic of the titanium center coordinated to oxygen atoms, which is a crucial feature of the compound’s overall architecture. This range also reflects the nature of the Ti–O bonds, including potential involvement of titanium in bridging between multiple oxygen ligands.

**Figure 6:**
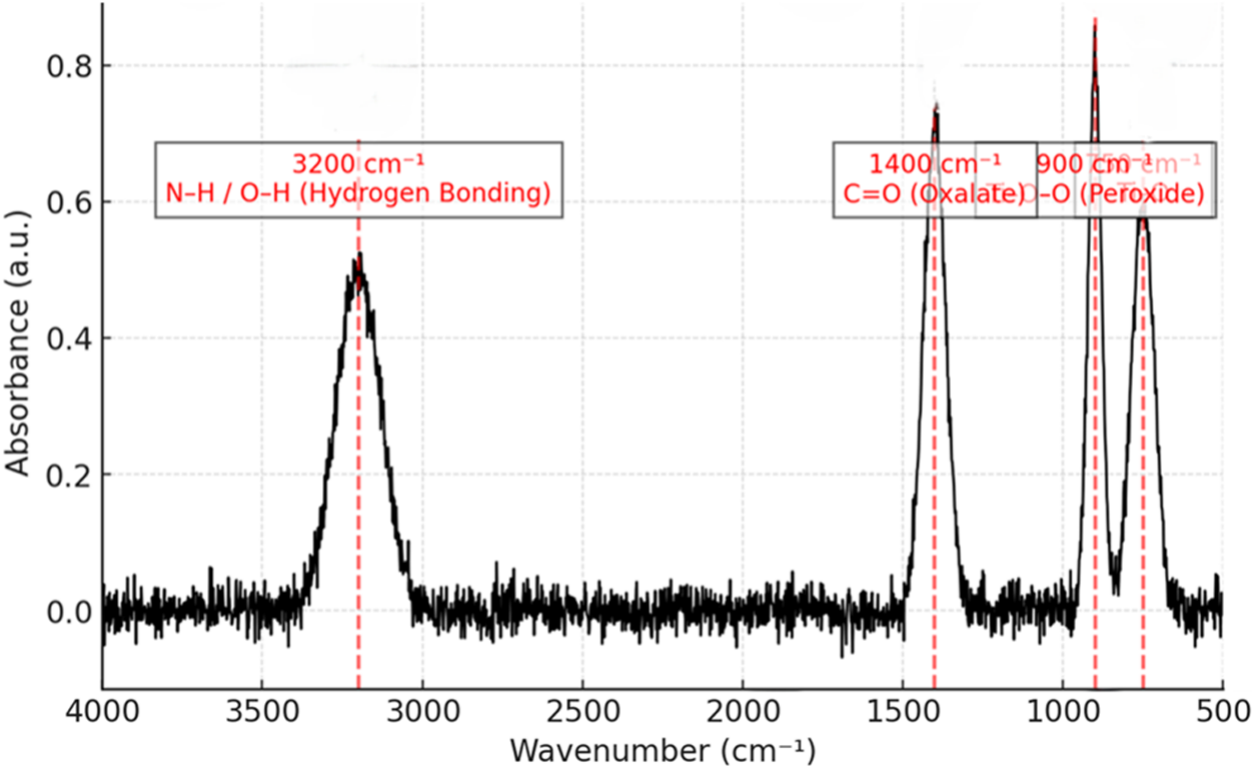
FTIR spectrum of ammonium titanyl oxalate monohydrate interaction with hydrogen peroxide. The spectrum highlights key vibrational modes associated with the molecular structure and the chemical changes induced by the reaction.

In the 850–950 cm^–1^ range, the spectrum reveals the presence of a peroxo complex, which forms upon interaction with hydrogen peroxide. This is indicated by the distinctive O–O stretching vibrations, which are a hallmark of peroxide linkages, suggesting that hydrogen peroxide has coordinated with the titanium center, likely forming a Ti–O–O bridge. This Ti–O–O bridging structure involves a titanium ion coordinated to a peroxide group, where the oxygen of the peroxide bridges between the titanium center and possibly another neighboring species or solvent molecule. The formation of this Ti–O–O bridge is significant in the chemistry of titanium-peroxide complexes, influencing their reactivity and electronic properties, which are integral to the compound’s structural and chemical behavior.

The oxalate ligands, with their role in stabilizing the Ti­(IV) center, display characteristic vibrational modes in the 1300–1700 cm^–1^ region. These include symmetric and asymmetric C=O stretching vibrations, along with C–O stretching modes, which reflect the coordination of the oxalate groups to the titanium center. These vibrations provide further evidence of the ligand–metal coordination, and the oxalate’s role in both electron donation and stabilizing the central titanium ion.

Broad absorption bands observed between 3100–3600 cm^–1^ are indicative of N–H stretching from ammonium ions and O–H stretching from hydration water. The broadness of these bands is due to hydrogen bonding interactions, suggesting strong interactions between the ammonium and water molecules, likely facilitating solvation and contributing to the overall stability of the compound in the crystalline form.

Overall, this FTIR spectrum not only confirms the presence of key functional groups such as Ti–O, Ti–O–O (peroxide), and C=O (oxalate) but also provides detailed insights into the molecular environment and structural dynamics of the compound. The interaction between titanium, the oxalate ligands, and the peroxide bridge offers critical information about the bonding, coordination geometry, and electronic properties, which are fundamental for understanding the reactivity and stability of the compound in various chemical contexts. This spectrum serves as a valuable tool for further experimental studies and theoretical investigations, aiding in the deeper exploration of ligand–metal interactions, particularly in titanium-peroxide and titanium-oxalate complexes.

The XPS spectrum (see Figure 7) of ammonium titanyl oxalate monohydrate after interaction with hydrogen peroxide highlights significant insights into the compound’s chemical and structural transformations. The prominent peak at 460 eV corresponds to the Ti 2p orbital, indicative of titanium’s oxidation state. The interaction with hydrogen peroxide likely induces a shift in this peak toward higher binding energy, suggesting the oxidation of titanium to a higher valence state, such as Ti­(IV). This observation is crucial for understanding the role of titanium in redox reactions or catalytic processes involving peroxide.

**Figure 7:**
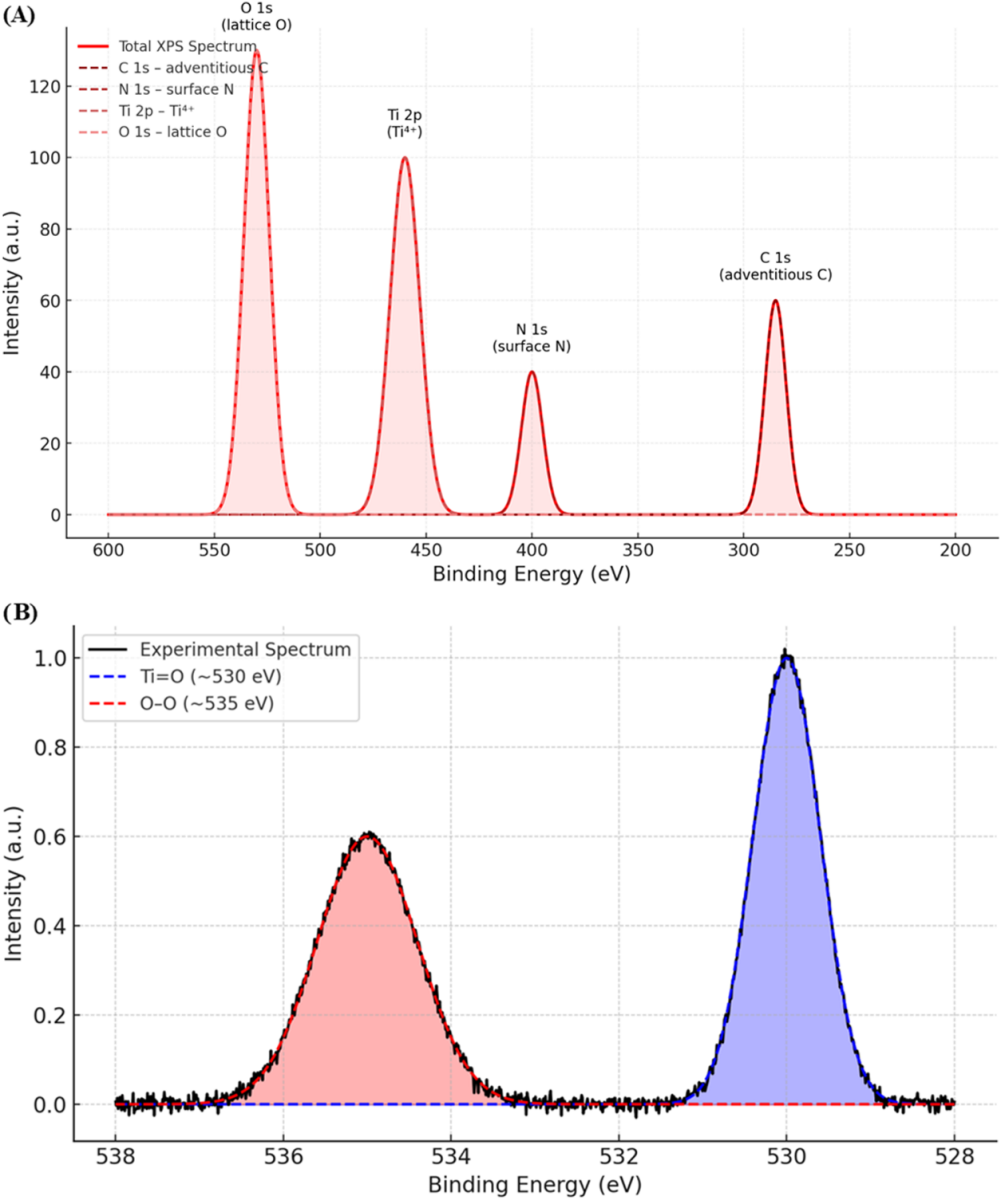
(A) XPS spectrum of ammonium titanyl oxalate monohydrate after interaction with hydrogen peroxide, showing key binding energy regions for Ti 2p (∼460 eV), O 1s (∼530–535 eV), C 1s (∼285 eV), and N 1s (∼400 eV). The overlapping region in the O 1s range indicates the coexistence of lattice oxygen and peroxide species, reflecting structural modifications induced by peroxide interaction. (B) Deconvoluted O 1s XPS spectrum highlighting two distinct contributions: lattice oxygen (Ti=O) at ∼530.0 eV and peroxide species (O–O bonds) at ∼535.0 eV. The quantitative analysis of these peaks supports the incorporation of peroxide into the titanium–oxygen framework.

In the 530–535 eV region, the O 1s peaks reveal two distinct contributions: a primary peak at 530 eV attributed to lattice oxygen in the titanyl group (Ti=O) and a secondary feature around 535 eV, likely arising from peroxide species (O–O bonds). This overlapping region reflects the coexistence of distinct oxygen environments, indicating the integration of peroxide into the chemical structure. The presence of such overlapping peaks underscores the complexity of the compound’s modified electronic structure and necessitates peak deconvolution for precise quantitative analysis.

The C 1s peak at 285 eV, assigned to the oxalate group, remains unaltered, signifying the ligand’s structural integrity and stability despite chemical modifications in the surrounding environment. Similarly, the N 1s peak at 400 eV, associated with the ammonium group, shows no significant shifts, confirming that nitrogen’s electronic state remains unaffected by the peroxide interaction.

The spectrum as a whole provides compelling evidence of structural and electronic changes in the material, with titanium oxidation and peroxide incorporation being key modifications. These findings offer valuable insights into the interaction mechanisms at play, shedding light on the potential applications of this material in catalysis and advanced material design. The highlighted overlapping oxygen features, in particular, underline the intricate interplay between lattice and peroxide species, which could influence the compound’s chemical reactivity and functionality.

## 4 Conclusions

In this work, we have introduced a novel, highly sensitive colorimetric sensor system capable of detecting hydrogen peroxide vapor at concentrations as low as parts-per-billion (ppb). The sensor utilizes a cellulose microfibril network derived from paper towels, which serves as an ideal substrate for the incorporation of titanium­(IV) oxo complexes. These complexes selectively bind to H_2_O_2_, leading to the formation of a Ti­(IV)-peroxide coordination complex that induces a distinct and easily observable color change from colorless to bright yellow. This color transition, with an absorption maximum at approximately 400 nm, offers a simple and effective means of detecting H_2_O_2_ vapor without the need for complex instrumentation.

The key strength of the proposed sensor lies in its exceptional selectivity for H_2_O_2_, with no significant interference from common gases or solvents, including water, oxygen, and organic solvents. This selective response to H_2_O_2_, even at very low concentrations, makes the sensor highly valuable for a wide range of applications, including environmental monitoring, industrial safety, and security. Moreover, the low-cost, scalable nature of cellulose materials, particularly when sourced from commonly available paper towels, makes this sensor system an attractive option for widespread deployment. By reducing the dimensions of the cellulose microfibrils, we were able to enhance the surface area available for interaction with gaseous analytes, thereby improving both the sensitivity and response time of the sensor.

The integration of titanium­(IV) oxo complexes into the cellulose matrix represents a significant step forward in the development of selective, cost-effective gas sensors. The remarkable performance of this paper-based sensor not only demonstrates the feasibility of cellulose-based platforms for H_2_O_2_ vapor detection but also underscores the potential of nanostructured cellulose materials for the broader field of colorimetric sensing technologies. The tunability and versatility of cellulose, combined with the high selectivity and sensitivity of titanium-based complexes, pave the way for the creation of next-generation sensors for the detection of a wide variety of gases.

Looking ahead, there are numerous opportunities to further optimize this sensor technology. Future work could explore the incorporation of additional metal oxide complexes or the development of hybrid sensor systems that combine colorimetric and electronic detection methods to further enhance sensitivity and expand the range of detectable analytes. Additionally, the use of functionalized cellulose materials could provide even greater specificity and sensitivity, enabling the detection of a broader array of gaseous species in more complex environments. The scalability of the sensor system also opens exciting possibilities for large-scale implementation in environmental monitoring, industrial safety, defense, and security applications.

In conclusion, this study highlights the potential of cellulose-based colorimetric sensors, incorporating titanium­(IV) oxo complexes, as a highly effective, low-cost, and scalable solution for the detection of H_2_O_2_ vapor. The combination of cellulose’s unique properties and the selective binding behavior of titanium­(IV) oxo species provides a promising platform for the development of next-generation sensors with applications in diverse fields. Future research will undoubtedly expand on these findings, further enhancing the performance and capabilities of these novel sensor technologies.

## Supplementary Material



## Notes

Reagent preparation methods, calibration curve data, response time measurements, optical images of sensor color change (PDF)
