# Supplementary material for: Designing Tunable Paper-Based Colorimetric Sensor for Precise Detection of Hydrogen Peroxide Vapor
Source: ACS Omega. 2025 Aug 1;10(31):34276–83. doi: 10.1021/acsomega.5c01380 (PMC12355273; doi:10.1021/acsomega.5c01380)
Supplement: Supplementary file 1 [file ao5c01380_si_001.pdf]

Designing Tunable Paper-Based Colorimetric Sensor for Precise Detection of Hydrogen Peroxide Vapor

Rayhan Hossain[1]*, Allen Apblett[2], and Nicholas F. Materer[2,]*

[1]Department of Natural Sciences, 107 Science Faculty Center, University of Michigan, Dearborn, Michigan 48128, USA
[2] Department of Chemistry, 316 Physical Science, Oklahoma State University, Stillwater, Oklahoma 74078, USA
***Correspondence: rayhan.hossain@okstate.edu and nicholas.materer@okstate.edu

Supporting Information:

UV-vis absorption titration of colorimetric reaction between titanyl salt and H_2O_2:

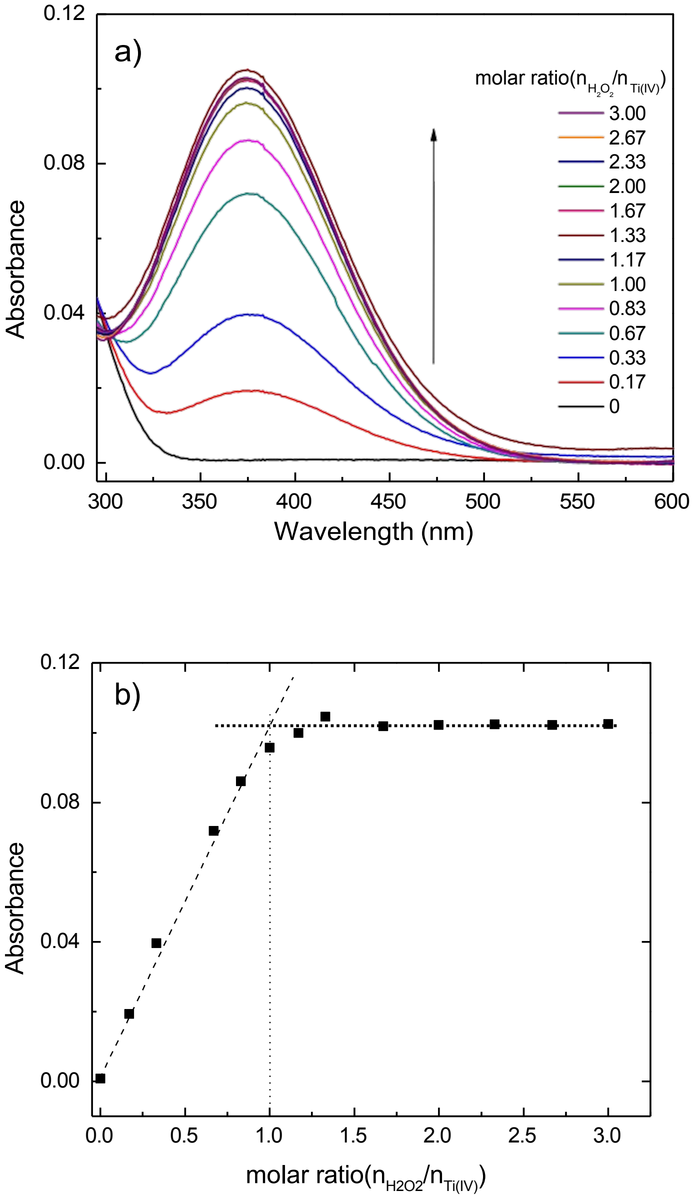

Figure S1. a) UV-vis absorption spectra of 1.2×10^{-4} mol/L aqueous solution of titanyl oxalate as measured with addition of different molar ratios of hydrogen peroxide solution. b) Absorbance measured at the maximum wavelength (400 nm) as a function of the molar ratio of H_2O_2/Ti(IV), for which a turning point around molar ratio of 1.0 indicates the 1:1 stoichiometric reaction between H_2O_2 and Ti(IV) salt as illustrated in Figure 1.

Selectivity test against potential interferences:

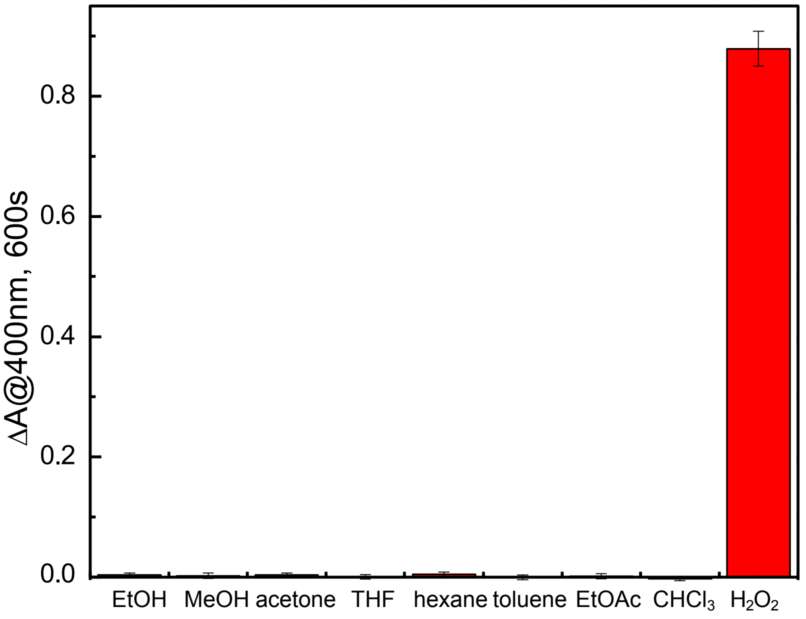

Figure S2. The increase of absorbance (ΔA) measured at 400 nm over the thin film of titanyl oxalate upon exposure to the saturated vapor of 35 wt % H_2O_2 solution (225.4 ppm), compared to the vapors of other common solvents: ethanol (89,000 ppm), methanol (131,000 ppm), acetone (260,000 ppm), THF (173,000 ppm), hexane (130,000 ppm), toluene (26,000 ppm), ethyl acetate (100,000 ppm), chloroform (140,000 ppm). The exposure time was 600 seconds. The exposure time was fixed at 600 seconds.
